# Optimization of a Microwave Polarimeter for Astronomy with Optical Correlation and Detection

**DOI:** 10.3390/s23052414

**Published:** 2023-02-22

**Authors:** Guillermo Pascual-Cisneros, Francisco J. Casas, Patricio Vielva

**Affiliations:** Instituto de Física de Cantabria (IFCA), Avda. Los Castros s/n, 39005 Santander, Spain

**Keywords:** instrumentation, Cosmic Microwave Background, polarization, microwave, photonics, astronomy, direct imaging, synthesized imaging, interferometry

## Abstract

Cosmic Microwave Background (CMB) B-modes detection is the main focus of future CMB experiments because of the valuable information it contains, particularly to probe the physics of the very early universe. For this reason, we have developed an optimized polarimeter demonstrator sensitive to the 10–20 GHz band in which the signal received by each antenna is modulated into a Near Infrared (NIR) laser by a Mach–Zehnder modulator. Then, these modulated signals are optically correlated and detected using photonic back-end modules consisting of voltage-controlled phase shifters, a 90-degree optical hybrid, a pair of lenses, and an NIR camera. During laboratory tests, a 1/f-like noise signal related to the low phase stability of the demonstrator has been found experimentally. To solve this issue, we have developed a calibration method that allows us to remove this noise in an actual experiment, until obtaining the required accuracy level in the measurement of polarization.

## 1. Introduction

The standard cosmological model postulates that our observed Universe has its origin in an exponential expansion, known as cosmic inflation, which grew a highly dense region of spacetime from quantum to macroscopic scales, drastically reducing the initial energy density of that region. The Universe resulting from this inflation is highly homogeneous and isotropic, and exhibits fluctuations in energy density that will give rise to the large-scale structure we observe, due to gravitational interaction. Prior to this structure formation, in the first instants, the inflationary potential decays in the formation of elementary particles, e.g., [1]. Thus, over a period of about 380,000 years, during which the Universe continues to expand (though no longer exponentially) and cool, radiation and matter form a plasma in which photons are constantly scattered by free electrons. It is after this period of time that the temperature is low enough to efficiently produce neutron hydrogen atoms, favoring the decoupling of radiation and matter, thus giving rise to a radiation background which today has a temperature of about 2.726 K [2], and is known as the Cosmic Microwave Background (CMB).

This CMB radiation was postulated by Gamow, Alpher, and Hernan in 1948 [3] and detected by Penzias and Wilson in 1964 [4]. Since then, the CMB has been an invaluable resource for studies in astrophysics and cosmology. Its intensity and polarization measurements provide information to test different cosmological models that define the physics of the early Universe, its energy/matter content, its evolution, and, eventually, its fate, e.g., [5].

Both space missions, e.g., [6,7,8] and ground-based experiments, e.g., [9,10,11], have been dedicated to measuring with increasing sensitivity, the temperature, and polarization anisotropies of the CMB, offering a set of discoveries on different fundamental questions in cosmology, such as the primordial spectrum of density perturbations, the geometry of the universe, its expansion rate, or its matter content, just to name a few, e.g., [5]. Nowadays, most of the experimental efforts are aligned with the ultimate goal of detecting the weak B-mode polarization signal, e.g., [12] predicted in the framework of cosmic inflation, as the imprint left by the primordial gravitational waves assumed to be produced during this early phase of exponential expansion of the Universe, e.g., [13]. In fact, the detection of the B-mode may allow us, in addition to corroborating cosmic inflation and characterizing the properties of its potential, to shed light on other aspects of fundamental physics such as the number of neutrino species and their masses; the existence of magnetic fields in the early Universe; the origin of magnetic fields in galaxies and galaxy clusters; the distribution of matter in the Universe, or the cosmic birefringence, e.g., [14].

The B-mode signal of the primordial CMB is extremely weak. The best current upper limits on its amplitude (expressed in terms of the *r* parameter of the scalar-tensor relation [15]) give r<0.032 with 95% confidence. Their detection is an instrumental and data analysis challenge. In fact, the signal is much weaker than the already known astrophysical microwave emissions generated in our Galaxy, mainly synchrotron radiation dominating at low frequencies (1–50 GHz) and thermal emission from dust dominating at high frequencies (300–1000 GHz). In addition, the B-modes of the primordial CMB are also contaminated by a weak lensing effect caused by the gravitational potential associated with the distribution of the large-scale structure of the cosmic web on the primordial radiation (e.g., Figure 35 in [16]). The above-mentioned difficulties in detecting the B-mode mean that experiments dedicated to its measurement have to rely on ultra-sensitive, low-noise instrumentation, and exquisite control of systematics.

CMB experiments usually operate with direct imaging instruments with detectors placed in the focal plane of a telescope. This means that the sensitivity is limited by the space available at the focal plane, i.e., by the number of receivers that can be placed, which is especially critical at low frequencies due to the relatively large size of the required antennas. As the sensitivity required for CMB experiments is increasing, and in particular for frequencies between 10 and 50 GHz, where antenna size remains a limiting factor, direct imaging telescopes are facing a certain limitation.

On the contrary, interferometric designs are not limited by space, in fact, resolution and sensitivity are only limited by the larger distance between antennas (baseline) and the number of baselines, respectively. Still, low-frequency CMB interferometers face another limitation, which is that they cannot afford more than a few tens of receivers due to limitations in phase control and routing of a high number of microwave signals. Therefore, new approaches are needed to improve the number of receivers and thus the sensitivity.

On the one hand, it is possible to use Field Programmable Gate Arrays (FPGAs) to create a digital correlator based on Michelson interferometry, but this approach does not seem the most suitable option, as the number of FPGAs scales with the number of baselines, which means that the cost of the experiment, power consumption and complexity scales as N(N−1)/2, where *N* is the number of antennas.

On the other hand, electro-optical modulators and simple optical configurations can be used to correlate the signal following a Fizzeau interferometry scheme that produces a final synthesized image. An electro-optical correlator prototype can already be found at [17].

In this paper, we present an optimized version of the interferometer prototype described in [17], which is made up of a front-end module (FEM) connected to an optical correlation and detection stage (OCDS) by means of a frequency up-conversion stage (FUS). Both, FUS and OCDS form the electro-optical back-end module (EOBEM).

The FEM consists of several 10–20 GHz bandwidth microwave receivers presenting a simplified design with respect to those of the QUIJOTE experiment [18,19]. The FUS is composed of a set of commercial LiNbO${}_{3}$ electro-optical Mach–Zehnder modulators (MZM) that modulate the FEM microwave signals in a 1550 nm laser. Finally, the OCDS is composed of a set of photonic circuits that are connected to a final optical stage. The photonic circuits are two phase shifters and an optical 90${}^{\circ }$ hybrid per receiver, which are in charge of modulating the polarization state in the same way as the former prototype’s microwave phase-switching and correlation modules, while the final optical stage is composed of a fiber array bundle, a pair of lenses, and a camera, which are in charge of interfering and detecting the signals, providing a final image.

The main advantage of this polarimeter version compared to the previous one [17] is that the frequency up-conversion is performed at an earlier stage, reducing the required number of MZMs by a factor of 2. This allows a significant reduction in cost, volume, weight, and power consumption as a consequence of moving the microwave phase switching and correlation modules to the electro-optical part of the polarimeter. Another advantage of this optimized version, as is discussed at the end of Section 4, is the possibility of implementation using Photonic Integrated Circuit (PIC) technology. This technology opens the door to further size reductions and better control of the systematics. In comparison with the microwave (MW) ICs, photonic components present the advantage of being able to manage diverse ultra-broad-band microwave signals easily because, after their conversion to the NIR frequency range, they all behave similar to narrow-band signals independently of the initial MW bandwidth. So, a given photonic component can be used for different MW frequency bands and bandwidths, while different MW ICs would be required in such cases.

Finally, we have implemented and tested a preliminary version of the optimized polarimeter to show its present performance and potential utility in experiments to characterize polarization in the low-frequency ranges of the CMB spectra.

This paper is organized as follows. After this introduction (Section 1), we provide a detailed description of the laboratory demonstrator, Section 2. Then, in Section 3, we show a theoretical model of the demonstrator. Afterwards, in Section 4 our laboratory measurements are presented. In Section 5, the demonstrator results and future improvements are discussed and, finally, the conclusions obtained from this work are presented in Section 6.

## 2. Laboratory Demonstrator Description

**Figure 1:**
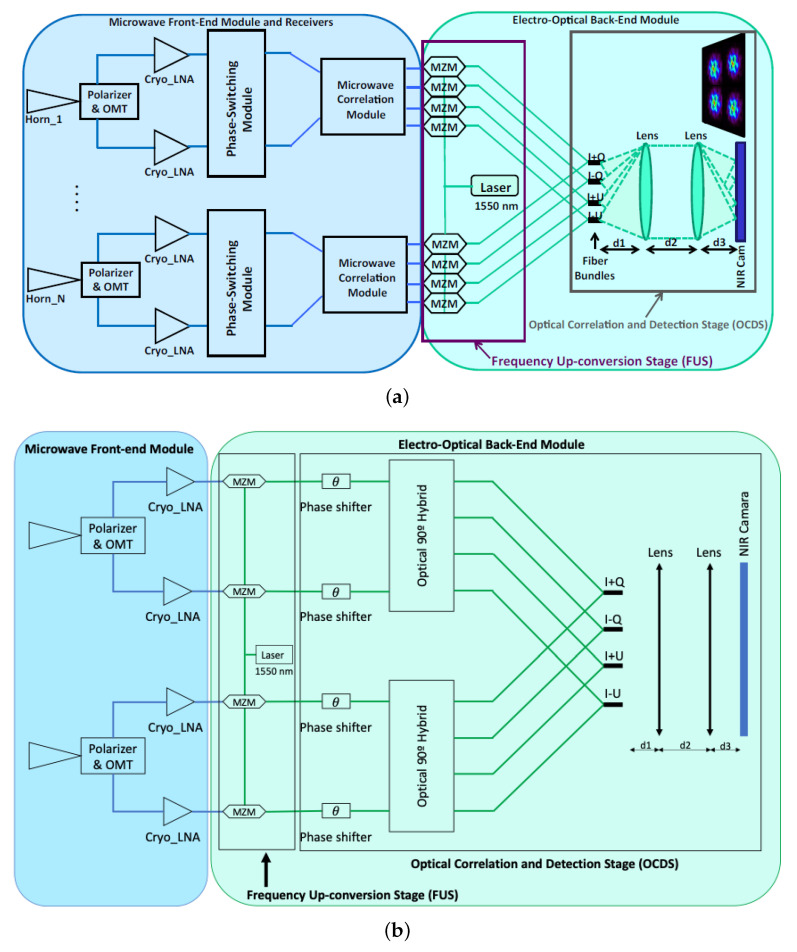
Simplified block diagram of the two microwave polarimeter for N receivers with near-infrared (1550 nm) correlation. (**a**) Microwave polarimeter previously proposed and reproduced with permission from F. J. Casas, Sensors; published by MDPI, 2019 [17]; (**b**) optimized version.

In this work, an optimized version of the interferometer prototype described in [17] is proposed. Both designs are shown in Figure 1: the previous model, Figure 1a, and the new proposed version, Figure 1b.

As discussed above, the optimized polarimeter shown in Figure 1b is composed of several microwave receivers (FEM) connected to an EOBEM divided into two main sections (the FUS and the OCDS). As in the former case, the system provides four different outputs proportional to different combinations of the Stokes parameters (I+Q, I−Q, I+U, I−U) per receiver. However, due to the presence of an optical phase shifter, it is possible to phase-modulate the hybrid inputs and then measure the full polarization state of the incoming signal at each of the outputs.

Most of the polarimeter components have already been described in [20] with the exception of the $90^{\circ }$ optical-hybrid and the phase-shifter. Nevertheless, the tested demonstrator is a simpler version of the proposed polarimeter: the operation of only one receiver is going to be tested which means that the polarimeter is operating as a direct image instrument. In this section, the simplified demonstrator is described in detail.

### 2.1. Front-End Module

In the original concept, the cryogenic receivers have the same conceptual design as those from the QUIJOTE experiment [18,19] covering the whole 10–20 GHz band.

**Figure 2:**
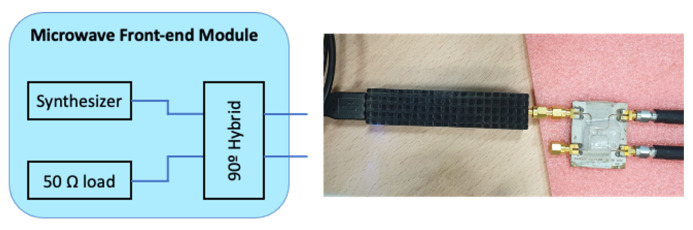
Block diagram of the front-end module used in the demonstrator. On the right, a scheme of the connected components is shown.

However, in our demonstrator, a 10 GHz single-frequency signal produced by Pasternack PE11S390 10–20 GHz frequency synthesizer is used. This signal is connected to the electro-optical modulators via a 90${}^{\circ }$ microwave hybrid with the other input connected to a 50 Ω load. This is a simplification that can be assumed because the two different hybrid outputs are equivalent to those produced by the original concept if the incident polarization angle is equal to 0${}^{\circ }$ (see Figure 2).

Since the receivers have been already tested in [17], here we can focus on the behavior of the demonstrator, by simply replacing them with the synthesizer and the 90${}^{\circ }$ hybrid described above.

### 2.2. Electro-Optical Back-End Module

The EOBEM is in charge of obtaining the polarization stages and correlating the final signal in the near-infrared camera. As it has been commented before, it is divided in two main stages: the FUS and the OCDS.

**Figure 3:**
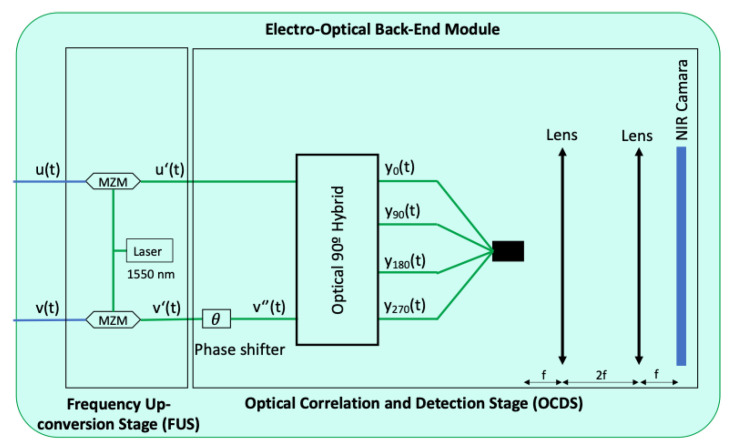
Block diagram of the electro-optical back-end module built on the lab.

Both the complete concept of the EOBEM, Figure 1b, and the simplified demonstrator’s one, Figure 3, are quite similar but, as mentioned above, only one receiver operating in direct imaging has been implemented for simplicity.

The demonstrator’s FUS is composed of two commercial Mach–Zehnder modulators (MZM), which are in charge of modulating the synthesizer input signal in a 1550 nm infrared laser. These MZM have a nominal bandwidth of 10 GHz, and they are of the same type already used in [17].

One of the most important elements in OCDS is the optical 90${}^{\circ }$ hybrid [21] because it provides four combinations of the two input signals that differ in the phase shifts applied to one of the inputs (0${}^{\circ }$, 90${}^{\circ }$, 180${}^{\circ }$, and 270${}^{\circ }$) and, therefore, it has a function similar to the one of the microwave correlation module in Figure 1a. In other words, as the inputs are defined by the polarization state of the input radiation, the optical 90${}^{\circ }$ hybrid outputs are proportional to given combinations of the Stokes’ parameters (I+Q, I−Q, I+U, and I−U). This implies that the polarization state of the input signal can be obtained by the simultaneous combination of the 4 measured output signals.

However, each output can be used to obtain an independent measurement just by adding a controlled phase-difference between the inputs. This phase-difference is produced in this work by two optical phase shifters [22] and they have the same function as the phase-switching module in Figure 1a, but in this case, the phase can be modulated in a continuous manner by means of a low-frequency control voltage.

Finally, there is a free-space optical system consisting of a fiber bundle, two lenses, and a camera, which correlate and detect the four outputs. An interesting point of this configuration is that the distance between the lenses will determine the operating mode of the polarimeter (direct imaging or synthesized interferometry) [17]. Another advantage is the possibility of adding optical filters in the focal plane of the first lens to improve sensitivity. As can be seen in Figure 3, a 4*f* configuration has been implemented in the demonstrator since, for simplicity, only one receiver has been implemented and only two out of the four outputs have been tested.

## 3. Demonstrator Model

In order to understand the behavior of the demonstrator we have developed a simple theoretical model. To simplify calculations, the input signal is treated as a complex single-frequency signal:

(1) $$y\left(t\right)=Ae^{i2\pi f_{m}t},$$

where *A* is the amplitude of the signal and $f_{m}$, the frequency of the microwave signal.

The microwave receivers in Figure 1b detect the polarized input signal, project it onto the polar axes, and combine both perpendicular projections by applying phase-shifts of $+90^{\circ }$ and $-90^{\circ }$ to different axes. Thus, following the notation in Figure 3, the output signals are defined as:

(2) $$\begin{array}{ccc} u(t) & = & \left(A_{x}+iA_{y}\right)e^{i2\pi f_{m}t},\\ v(t) & = & \left(A_{y}+iA_{x}\right)e^{i2\pi f_{m}t}. \end{array}$$

The polarization components, $A_{x}$ and $A_{y}$, are given by:

(3) $$\begin{array}{ccc} A_{x} & = & A\cos\phi ,\\ A_{y} & = & A\sin\phi ; \end{array}$$

where ϕ is the polarization angle. Note that when the polarization angle is $0^{\circ }$, the output is the same as that obtained by the front-end module of the demonstrator described above (see Figure 2).

Therefore, substituting (3) in (2), we obtain:

(4) $$\begin{array}{ccc} u(t) & = & Ae^{i2\pi f_{m}t}e^{i\phi },\\ v(t) & = & Ae^{i2\pi f_{m}t}ie^{-i\phi }. \end{array}$$

This signal is now modulated by the MZMs (frequency up-conversion stage). The simplified model assumed in the following equation to describe the behavior of the MZMs only takes into account the two main modulated side-bands and the optical carrier:

(5) $$u^{\prime }\left(t\right)=u\left(t\right)\frac{e^{i2\pi f_{c}t}+e^{-i2\pi f_{c}t}}{2}+ke^{i2\pi f_{c}t},$$

where $f_{c}$ is the frequency of the optical carrier and *k* is the attenuation of the optical carrier band. From this model, the infrared-modulated signals can be easily obtained as:

(6) $$\begin{array}{ccc} u^{\prime }\left(t\right) & = & \frac{A}{2}\left(e^{i2\pi (f_{m}+f_{c})t}+e^{i2\pi (f_{m}-f_{c})t}\right)e^{i\phi }+ke^{i2\pi f_{c}t},\\ v^{\prime }\left(t\right) & = & \frac{A}{2}\left(e^{i2\pi (f_{m}+f_{c})t}+e^{i2\pi (f_{m}-f_{c})t}\right)ie^{-i\phi }+ke^{i2\pi f_{c}t}. \end{array}$$

At this point, a term of the form $e^{i\theta (t)}$ is added to Equation (6), where the θ(t) function gives information about the phase modulation:

(7) $$\begin{array}{ccc} u^{\prime }\left(t\right) & = & \frac{A}{2}\left(e^{i2\pi (f_{m}+f_{c})t}+e^{i2\pi (f_{m}-f_{c})t}\right)e^{i\phi }+ke^{i2\pi f_{c}t},\\ v^{\prime \prime }\left(t\right) & = & \frac{A}{2}\left(e^{i2\pi (f_{m}+f_{c})t}+e^{i2\pi (f_{m}-f_{c})t}\right)ie^{i(\theta (t)-\phi )}+ke^{i2\pi f_{c}t}e^{i\theta (t)}. \end{array}$$

From now on, it is only necessary to add the effect of the optical hybrid of $90^{\circ }$ to obtain the final signals. It can be easily modeled using the following equations:

(8) $$\begin{array}{ccc} y_{0}\left(t\right) & = & u^{\prime }\left(t\right)+v^{\prime \prime }\left(t\right)e^{-i\pi /2},\\ y_{90}\left(t\right) & = & u^{\prime }\left(t\right)e^{-i\pi /2}+v^{\prime \prime }\left(t\right)e^{-i\pi /2},\\ y_{180}\left(t\right) & = & u^{\prime }\left(t\right)e^{-i\pi /2}+v^{\prime \prime }\left(t\right),\\ y_{270}\left(t\right) & = & u^{\prime }\left(t\right)e^{i\pi }+v^{\prime \prime }\left(t\right). \end{array}$$

Hence, substituting Equations (7) in (8), the hybrid output signals are given by:

(9) $$\begin{array}{ccc} y_{0}\left(t\right) & = & \frac{A}{2}\left(e^{i2\pi (f_{m}+f_{c})t}+e^{i2\pi (f_{m}-f_{c})t}\right)\left(e^{i\phi }+e^{i(\theta (t)-\phi )}\right)+ke^{i2\pi f_{c}t}\left(1-ie^{i\theta (t)}\right),\\ y_{90}\left(t\right) & = & -\frac{A}{2}\left(e^{i2\pi (f_{m}+f_{c})t}+e^{i2\pi (f_{m}-f_{c})t}\right)\left(e^{i(\theta (t)-\phi )}-ie^{i\phi }\right)-ike^{i2\pi f_{c}t}\left(1+e^{i\theta (t)}\right),\\ y_{180}\left(t\right) & = & i\frac{A}{2}\left(e^{i2\pi (f_{m}+f_{c})t}+e^{i2\pi (f_{m}-f_{c})t}\right)\left(e^{i(\theta (t)-\phi )}-e^{i\phi }\right)+ke^{i2\pi f_{c}t}\left(e^{i\theta (t)}-i\right),\\ y_{270}\left(t\right) & = & -\frac{A}{2}\left(e^{i2\pi (f_{m}+f_{c})t}+e^{i2\pi (f_{m}-f_{c})t}\right)\left(e^{i\phi }-ie^{i(\theta (t)-\phi )}\right)+ke^{i2\pi f_{c}t}\left(e^{i\theta (t)}-1\right). \end{array}$$

The next step is to model the image obtained on the camera through the square modulus of the output signals. However, since |A+B|≤|A|+|B|, it is only possible to obtain analytical results when k=0, or, in other words, when there is no optical carrier in the modulated signal, which is the ideal situation when operating the polarimeter. In this case, the detected signals are:

(10) $$\begin{array}{ccc} {\left|y_{0}\left(t\right)\right|}^{2} & = & \frac{A^{2}}{2}\cos^{2}\left(2\pi f_{c}t\right)\left[1+\cos\left(\theta (t)-2\phi \right)\right],\\ {\left|y_{90}\left(t\right)\right|}^{2} & = & \frac{A^{2}}{2}\cos^{2}\left(2\pi f_{c}t\right)\left[1+\cos\left(\theta (t)-(2\phi -\pi /2)\right)\right],\\ {\left|y_{180}\left(t\right)\right|}^{2} & = & \frac{A^{2}}{2}\cos^{2}\left(2\pi f_{c}t\right)\left[1+\cos\left(\theta (t)-(2\phi -\pi )\right)\right],\\ {\left|y_{270}\left(t\right)\right|}^{2} & = & \frac{A^{2}}{2}\cos^{2}\left(2\pi f_{c}t\right)\left[1+\cos\left(\theta (t)-(2\phi -3\pi /2)\right)\right]. \end{array}$$

Afterwards, as the camera is not able to sample signals with a frequency as high as $f_{c}$, in practice, what will be obtained is an average value measured over the sampling period ($T_{m}$). A good approximation to this situation is to replace the term $\cos^{2}\left(2\pi f_{c}t\right)$ in Equation (10) by 1/2, which is its average value over the sampling period ($\langle \cos^{2}\left(2\pi f_{c}t\right)\rangle =\int_{0}^{T_{c}}\cos^{2}\left(2\pi f_{c}t\right)\partial t=\frac{1}{2}$). Therefore, the signals detected by the camera are given as:

(11) $$\begin{array}{ccc} {\left|y_{0}\left(t\right)\right|}^{2} & = & \frac{A^{2}}{4}\left[1+\cos\left(\theta (t)-2\phi )\right)\right],\\ {\left|y_{90}\left(t\right)\right|}^{2} & = & \frac{A^{2}}{4}\left[1+\cos\left(\theta (t)-(2\phi -\pi /2)\right)\right],\\ {\left|y_{180}\left(t\right)\right|}^{2} & = & \frac{A^{2}}{4}\left[1+\cos\left(\theta (t)-(2\phi -\pi )\right)\right],\\ {\left|y_{270}\left(t\right)\right|}^{2} & = & \frac{A^{2}}{4}\left[1+\cos\left(\theta (t)-(2\phi -3\pi /2)\right)\right]. \end{array}$$

These expressions easily show how the polarimeter works and why the application of auxiliary phase modulation, provided in our case by the optical phase shifters, is important. In particular, if there is no auxiliary phase modulation, the combinations between the outputs will give the polarization state of the input signal, but the measurement will be greatly affected by the systematic errors of the polarimeter. However, when auxiliary phase modulation is applied, the phase of each output signal will determine the polarization state providing, at the same time, information about the instrumental errors that are affecting the measurement [23].

## 4. Polarimeter Demonstrator Characterization

**Figure 4:**
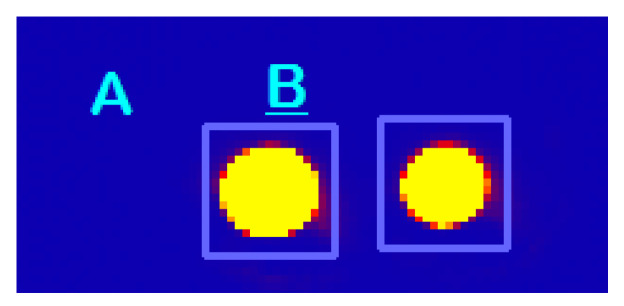
Example of the image captured by the camera. Two squares (A and B) delimiting the integration area around the IR beams can also be seen.

Once the demonstrator has been built and the active optical components have been stabilized, it is possible to perform the first direct measurements by integrating the mean value of the intensity detected by the camera in squares containing the image of each of the hybrid outputs (see Figure 4).

**Figure 5:**
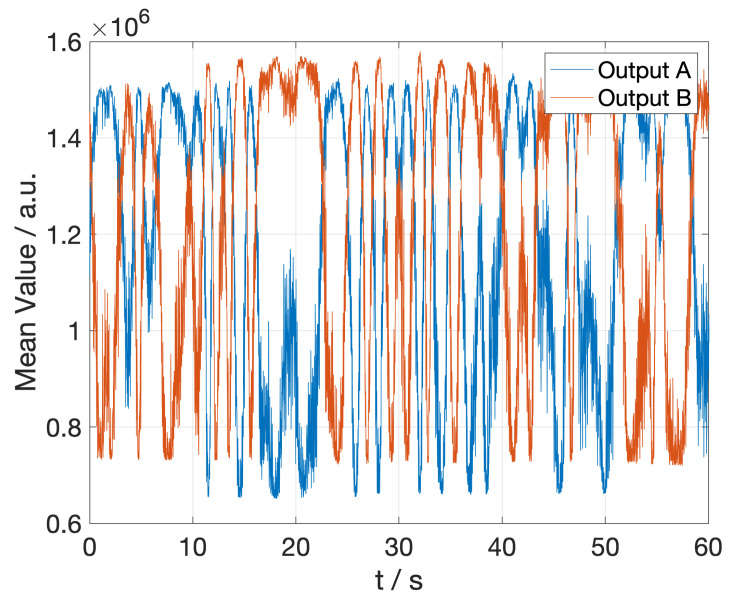
Demonstrator measurement obtained without any external phase modulation.

The optical phase shifters are switched off for the first tests, so the average value detected by the camera at each connected output should remain constant over time. However, as can be seen in Figure 5, there are slow random variations over time. These random variations are also present if the hybrid is connected directly to the laser (without any kind of modulation), so there must be some optical path variations (phase-shifts) in the demonstrator that can be caused by vibrations, atmospheric pressure, and temperature or fiber junctions, among others, so there is no easy way to suppress this noise.

**Figure 6:**
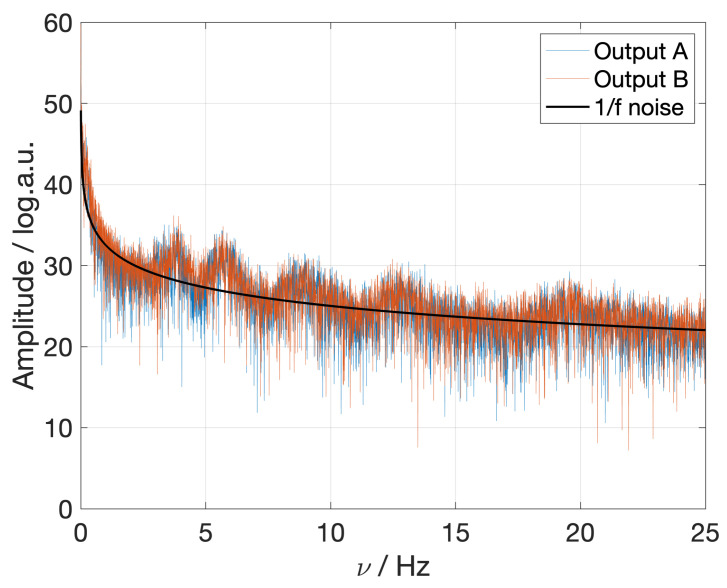
Fast Fourier Transform (FFT) of the measured signal obtained without external phase modulation. The black line represents a 1/*f* noise, as an illustration. The amplitude units correspond to $10\log_{10}\left(A\right)$ where *A* is the FFT of the amplitude.

This noise is quite similar to a 1/f noise with some additional components at higher frequencies (see Figure 6). However, these high frequency signals represent vibrations that may come from elements of the system that are not perfectly stabilized and could be, in principle, easily corrected.

As the frequency range of this noise signal is the same as the one provided by the external polarization modulators (the optical phase shifters), no further tests to measure microwave polarization were performed until a solution to the reported problem is found. Unfortunately, this circumstance has prevent us of doing direct comparisons between polarization measurements with the new and the previous version of the polarimeter. In a future work, we will implement a phase-shifting optical module with an appropriate response speed (time constants of the order of msec), allowing us to perform such measurements thanks to the application of the calibration method explained below.

## 5. Proposed Solution

As previously discussed, it is necessary to develop a methodology to reduce the random noise found in our demonstrator. A first solution would be to modulate the phase of the system faster than the natural frequency of the noise. Using the theoretical model shown in Section 3, we can explore whether fast modulation is sufficient to decouple the noise.

The function θ(t) previously introduced on Equation (11) serves to add the slow random term that was experimentally measured:

(12) θ(t)=νt+rand(t),

where ν is the phase modulation frequency. If ν is larger than the natural frequency of the random phase noise, then, for a small interval of time (e.g., $t^{*}\in \left[0,3/\nu \right]$), the noise can be considered as a constant free parameter (*r*), which couples to the phase and, afterwards, it couples to the polarization state:

(13) $$\begin{array}{ccc} {\left|y_{0}\left(t^{*}\right)\right|}^{2} & = & \frac{A^{2}}{4}\left[1+\cos\left(\nu t^{*}-\left(2\phi -r\right)\right)\right],\\ {\left|y_{90}\left(t^{*}\right)\right|}^{2} & = & \frac{A^{2}}{4}\left[1+\cos\left(\nu t^{*}-\left(2\phi -\pi /2-r\right)\right)\right],\\ {\left|y_{180}\left(t^{*}\right)\right|}^{2} & = & \frac{A^{2}}{4}\left[1+\cos\left(\nu t^{*}-\left(2\phi -\pi -r\right)\right)\right],\\ {\left|y_{270}\left(t^{*}\right)\right|}^{2} & = & \frac{A^{2}}{4}\left[1+\cos\left(\nu t^{*}-\left(2\phi -3\pi /2-r\right)\right)\right]. \end{array}$$

Therefore, in order to obtain the polarization state by performing fast phase modulation, it is necessary to know the value of the random phase noise in the corresponding interval.

Taking this into account, we have performed a calibration test using a perfectly known polarized signal, allowing us, in particular, to characterize the phase-noise in real time. Obviously, in our case, this is an artificial source whose properties are known with the precision required for the particular experiment.

To test this idea, a Matlab-based simulation has been used. The simulation considers a $0^{\circ }$ polarized input signal at 10 GHz, which is modulated following the theoretical scheme presented above (Equation (9), the simulation is also performed considering k=0 for simplicity but it can be adapted for different values). The phase is then modulated and the previously measured phase noise is added to the simulation.

The phase-modulation frequency is set to 3 GHz to reduce the computational cost of the simulation, since the simulation sampling time is $2\times 10^{-13}$ s (also reduced for the same reason). In a real experiment, the phase modulation frequency would be in the KHz range.

**Figure 7:**
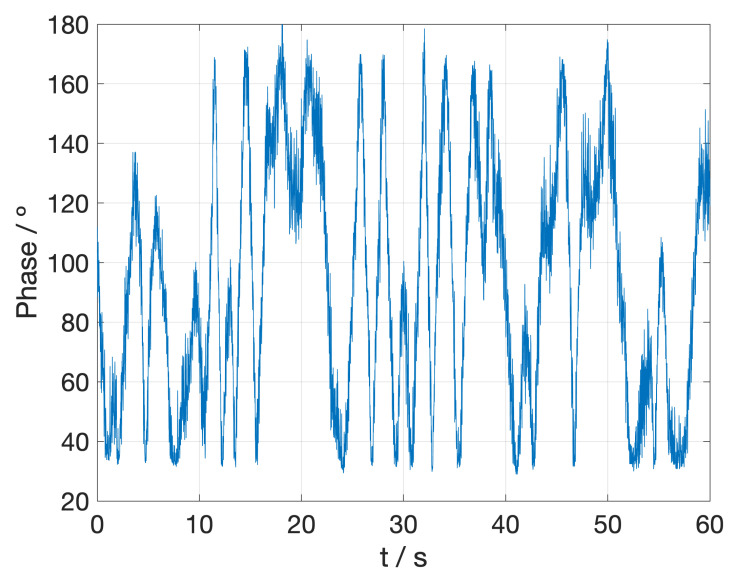
Phase noise extracted from the signal measured by the demonstrator. Time intervals have been modified to make simulation affordable.

The phase noise is obtained by subtracting the two measured signals in Figure 5, normalizing the result and finally calculating its arc-cosine (Figure 7). Again, the time interval has been drastically reduced to make the simulation affordable and the noise has been low-pass filtered, as the high-frequency peaks are expected to come from laser and modulation instabilities that can be corrected for. This leads to a slow noise whose maximum frequency is about 10 MHz.

Note that the modulation frequency is three orders of magnitude higher than the maximum noise frequency, similar to the factor expected in a real experiment, where the maximum noise frequency is close to 1 Hz and the modulation frequency is in the KHz range.

Once the phase noise has been added to the simulation, the square modulus of the $90^{\circ }$ optical hybrid outputs is obtained. The simulation measures the phase for 1 ns at each output and then waits for a user-defined time. The idea is to characterize the phase noise (value of *r* in Equation (13)) at several points and then try to reconstruct the original signal, modifying the waiting time so that it is as long as possible. In a real experiment, this waiting time is related to the time that can be used to measure the sky, so it is important to maximize it while obtaining the required accuracy in the noise characterization.

The measurements are simulated by obtaining the FFT of the entire 1 ns time period and calculating the phase at the 3 GHz peak. Then, this phase has to be modified according to the behavior of each output of the hybrid (Equation (13)) and, finally, the calculated phase noise is fitted by Fourier series and compared with the measured one (Figure 7).

**Figure 8:**
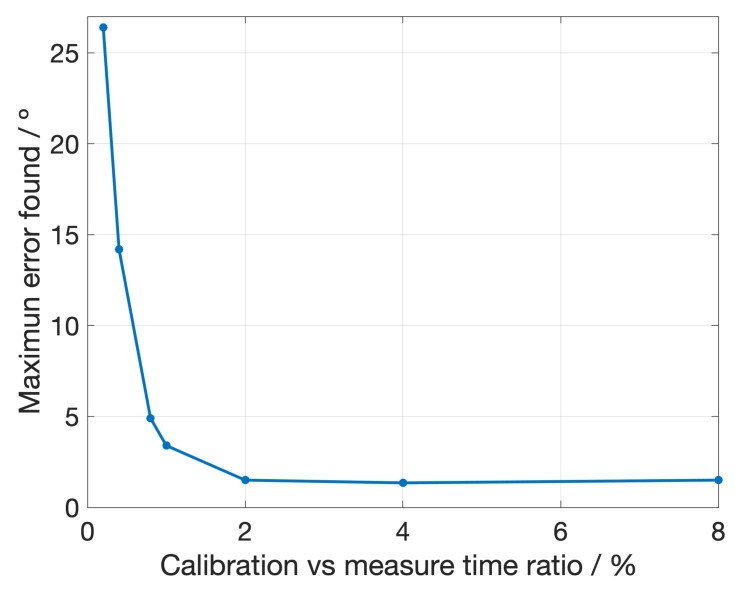
Maximum error found after applying our calibration method for different ratios between the time used for calibration measurements and the time used for sky ones.

**Figure 9:**
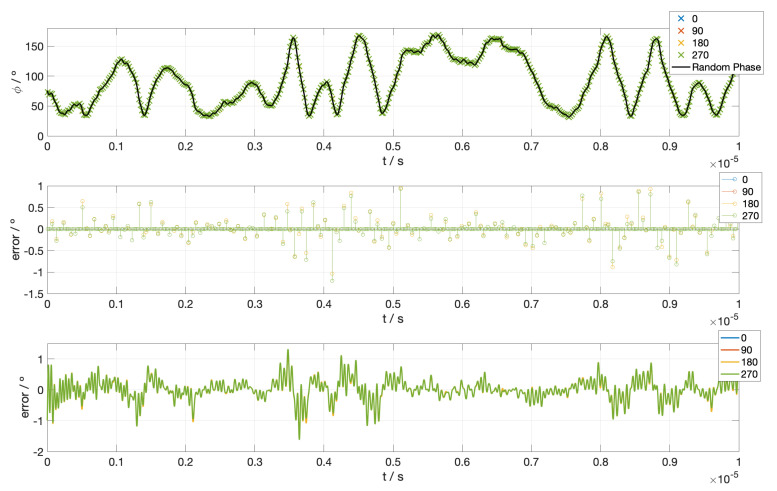
In the top panel, we show the demonstrator phase noise, black line, and the measured points for each output of the $90^{\circ }$ optical hybrid and its settings. The middle panel shows the residuals between the measured points and the demonstrator noise for each hybrid output. Finally, the residuals between the reconstructed noise by fitting those measured points with Fourier series and demonstrator noise can be seen in the lower panel for each hybrid output.

In Figure 8, we can see the maximum error reported on the phase (polarization angle) measurement after applying the described calibration method with different interval times between calibration measures. We can see that, as expected, the error decreases with the time used for calibration, but as the increase in the sensitivity is very small after a certain point, we can conclude that an optimal strategy is to use only a 5% of the total measuring time to calibrate the experiment, which means that the simulation timeout is 20 ns. The results obtained for this case are shown in Figure 9, where we can see the measured random phase noise and its characterization, with the corresponding residuals.

The largest error found in the fit is about $1.5^{\circ }$, which means that the largest error in the polarization angle is about $0.75^{\circ }$ (half of the phase error). However, this error is very much influenced by the accuracy of the calibration measurements, almost all values are close to $0^{\circ }$ error but some of them show fluctuations.

Moreover, $0.75^{\circ }$ is near the acceptable error for actual CMB experiments. In such experiments, it is expected to obtain lower error values than in the demonstrator case, due to the much slower phase errors derived from a more stabilized and controlled operation of all components. This would allow also to obtain a higher ratio between error frequency and phase modulation. Therefore, this methodology can be used to overcome random phase problems in a real CMB experiment.

## 6. Discussion and Conclusions

In this work, an optimized version of the polarimeter shown in [17] has been presented as a promising idea that can reduce the cost, size, and power consumption of the polarimeter. However, a 1/f type noise related to the phase of the system coupled to the polarization information has been found. Those phase errors can be up to 180 degrees in a typical polarization measurement.

To solve this issue, a method to characterize the error in real time has been provided and tested by simulations. It has been shown that in a real experiment, its effect can be partially eliminated, up to a good level of accuracy in the polarization measurement. A maximum remaining error of 0.75 degrees in the polarization angle has been proved, which is near the acceptable error level in experiments for the characterization of foreground signals (such as synchrotron) that are dominant in the low-frequency range of the CMB spectra. In actual experiments, lower error levels are expected, because the operation on all components should be more stabilized and controlled, in terms of pressure and temperature variations, than in our demonstrator.

On the other hand, their implementation on InP Photonic Integrated Circuits (PICs) represents a very interesting solution although no problems were encountered in the demonstrator here explored. Their small size, low power consumption, and fabrication method on large multi-project wafers make PICs interesting for experiments with a large number of detectors, such as ground-based CMB experiments at frequencies around 40 GHz.

These characteristics are also interesting in our case, as the waveguides are fixed and it is easier to stabilize temperature and pressure in small chips than in long fiber optic systems. Therefore, phase noise is expected to be drastically reduced.

**Figure 10:**
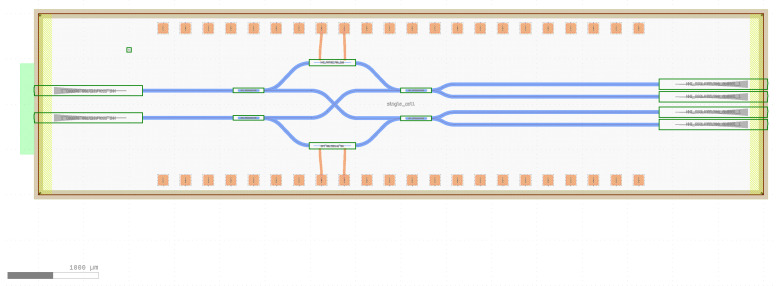
Design of a 8 × 2 mm photonic integrated circuit containing a $90^{\circ }$ hybrid.

In a future work, a PIC version of the proposed polarimeter will be designed and tested using dedicated electro-optical simulation software engines. In Figure 10, a preliminary design of the requested $90^{\circ }$ optical hybrid in PIC technology based on the [24] model is presented. It has been obtained using the BRIGHT Photonics Nazca Design software [25] with the Fraunhofer Institute HHI toolkit [26].

## Data Availability

For the readers interested in the experimental data related to this work, please contact to pascual@ifca.unican.es or casas@ifca.unican.es.

## Abbreviations

The following abbreviations are used in this manuscript:

CMB	Cosmic Microwave Background
MZM	Mach–Zehnder Modulators
FEM	Front-End Module
EOBEM	Electro-Optical Back-End Module
FUS	Frequency Up-conversion Stage
OCDS	Optical Correlation and Detection Stage
PIC	Photonic Integrated Circuits

## References

[1] Liddle A.R., Lyth D.H. (2000). Cosmological Inflation and Large-Scale Structure.

[2] Fixsen D.J. (2009). The Temperature of the Cosmic Microwave Background. Astrophys. J..

[3] Alpher R.A., Bethe H., Gamow G. (1948). The Origin of Chemical Elements. Phys. Rev..

[4] Penzias A.A., Wilson R.W. (1965). A Measurement of Excess Antenna Temperature at 4080 Mc/s. Astrophys. J..

[5] Aghanim N., Akrami Y., Ashdown M., Aumont J., Baccigalupi C., Ballardini M., Banday A.J., Barreiro R.B., Bartolo N., Planck Collaboration (2020). Planck 2018 results. VI. Cosmological parameters. Astron. Astrophys..

[6] Boggess N.W. (1992). The Cosmic Background Explorer (COBE): Mission and Science Overview. Highlights Astron..

[7] Jarosik N., Bennett C., Dunkley J., Gold B., Greason M., Halpern M., Hill R., Hinshaw G., Kogut A., Komatsu E. (2011). Seven-year wilkinson microwave anisotropy probe (WMAP*) observations: Sky maps, systematic errors, and basic results. Astrophys. J. Suppl. Ser..

[8] Ade P.A., Aghanim N., Arnaud M., Ashdown M., Aumont J., Baccigalupi C., Baker M., Balbi A., Banday A., Barreiro R. (2011). Planck early results. I. The Planck mission. Astron. Astrophys..

[9] Buder I. (2010). Q/U Imaging Experiment (QUIET): A ground-based probe of cosmic microwave background polarization. Millimeter, Submillimeter, and Far-Infrared Detectors and Instrumentation for Astronomy V.

[10] Barkats D., Aikin R., Bischoff C., Buder I., Kaufman J., Keating B., Kovac J.M., Su M., Ade P., Battle J. (2014). Degree-scale cosmic microwave background polarization measurements from three years of BICEP1 data. Astrophys. J..

[11] Ade P.A., Aikin R., Barkats D., Benton S., Bischoff C., Bock J., Brevik J., Buder I., Bullock E., Dowell C. (2014). Detection of B-mode polarization at degree angular scales by BICEP2. Phys. Rev. Lett..

[12] Hu W., White M. (1997). A CMB polarization primer. New Astron..

[13] Kamionkowski M., Kovetz E.D. (2016). The Quest for B Modes from Inflationary Gravitational Waves. Annu. Rev. Astron. Astrophys..

[14] Komatsu E. (2022). New physics from the polarized light of the cosmic microwave background. Nat. Rev. Phys..

[15] Tristram M., Banday A.J., Górski K.M., Keskitalo R., Lawrence C.R., Andersen K.J., Barreiro R.B., Borrill J., Colombo L.P.L., Eriksen H.K. (2022). Improved limits on the tensor-to-scalar ratio using BICEP and Planck data. Phys. Rev. D.

[16] Akrami Y., Ashdown M., Aumont J., Baccigalupi C., Ballardini M., Banday A.J., Barreiro R.B., Bartolo N., Basak S., Benabed K. (2020). Planck2018 results. Astron. Astrophys..

[17] Casas F.J., Ortiz D., Aja B., de la Fuente L., Artal E., Ruiz R., Mirapeix J.M. (2019). A Microwave Polarimeter Demonstrator for Astronomy with Near-Infra-Red Up-Conversion for Optical Correlation and Detection. Sensors.

[18] Rubiño-Martín J.A., Rebolo R., Aguiar M., Génova-Santos R., Gómez-Reñasco F., Herreros J., Hoyland R., López-Caraballo C., Santos A.P., de La Rosa V.S. (2012). The QUIJOTE-CMB experiment: Studying the polarisation of the galactic and cosmological microwave emissions. Ground-Based and Airborne Telescopes IV.

[19] López-Caniego M., Rebolo R., Aguiar M., Génova-Santos R., Gómez-Reñasco F., Gutierrez C., Herreros J., Hoyland R., López-Caraballo C., Santos A. (2014). The QUIJOTE CMB Experiment: Status and first results with the multi-frequency instrument. arXiv.

[20] Ortiz D., Casas F.J., Ruiz-Lombera R., Mirapeix J. (2017). Electro-optic correlator for large-format microwave interferometry: Up-conversion and correlation stages performance analysis. Rev. Sci. Instruments.

[21] Kylia COH24 90º Hybrid. https://kylia.com/90-hybrids.

[22] Phoenix Photonics Fiber Optic Phase Shifter. http://www.phoenixphotonics.com/website/products/fiber-phase-shifter.html.

[23] Casas F.J., Vielva P., Barreiro R.B., Martínez-González E., Pascual-Cisneros G. (2022). Polarization Calibration of a Microwave Polarimeter with Near-Infrared Up-Conversion for Optical Correlation and Detection. Sensors.

[24] Kunkel R., Bach H.G., Hoffmann D., Weinert C., Molina-Fernandez I., Halir R. First monolithic InP-based 90°-hybrid OEIC comprising balanced detectors for 100GE coherent frontends. Proceedings of the 2009 IEEE International Conference on Indium Phosphide Related Materials.

[25] BRIGHT Photonics Nazca Design Software. https://nazca-design.org/.

[26] Fraunhofer Heinrich Hertz Institute. https://www.hhi.fraunhofer.de/.

